# Community-based postnatal care model: Catalyst for management of mothers and neonates

**DOI:** 10.4102/curationis.v47i1.2563

**Published:** 2024-04-22

**Authors:** Katekani J. Shirindza, Thivhulawi Malwela, Sonto M. Maputle

**Affiliations:** 1 Department of Advanced Nursing Science, Faculty of Health Sciences, University of Venda, Thohoyandou, South Africa

**Keywords:** catalyst, management, model, neonates, postnatal women

## Abstract

**Background:**

Early postnatal discharge is perceived as a factor that contributes to the possibilities of the maternal, neonatal complications and deaths. The implementation of the community-based postnatal care model is crucial to mitigate the morbidity and mortality of postnatal women and neonates during the first weeks of delivery. A community-based postnatal care model was developed for the management of neonates during the postnatal care period in the community.

**Objectives:**

The study aims to share the developed community-based postnatal care model that could assist postnatal women in the management of neonates.

**Method:**

Empirical findings from the main study formed the basis for model development. The model development in this study was informed by the work of Walker and Avant; Chinn and Kramer Dickoff, James and Wiedenbach; and Chinn and Jacobs.

**Results:**

The results indicated that there was no community-based postnatal care model developed to manage neonates. The model is described using the practice theory of Dickoff, James and Wiedenbach elements of agents, recipients, context, process, dynamics and outcomes within the community context of the postnatal care period. The model was further described by Chinn and Krammer following the assumptions of the model, concept definition, relation statement and nature of structure.

**Conclusion:**

The utilisation of the model is critical and facilitates the provision of an enabling and supportive community-based context by primary caregivers for the effective management of neonates.

**Contribution:**

This study provides a reference guide in the provision of community-based postnatal care by postnatal women after discharge from healthcare facilities.

## Introduction

The first 28 days of life – the neonatal period is the most vulnerable time for a child’s survival (Owusu et al. 2018). Globally, neonatal mortality has seen a downward trend in recent years (Dos Santos et al. 2011). During this period, nearly half of the neonates’s deaths occurred as a result of early discharge from the healthcare facilities (Rao et al. 2021). However, the decline in neonatal deaths has been slower in 2015, resulting in 2.4 million neonatal deaths (Rhoda et al. 2018). Sub-Saharan Africa had the highest neonatal deaths (43%), followed by central and Southern Asia with 36% of neonatal deaths. South Africa had 70% of neonatal deaths in 2018 (Akombi & Renzaho 2019).

According to Velaphi and Rhouda (2012), South Africa is one of the countries in which neonatal mortality has increased over the last 20 years. The major causes of neonatal deaths are related to prematurity and intrapartum hypoxia. Several interventions have been shown to reduce neonatal deaths, and if implemented on a wider scale, they could reduce neonatal deaths significantly. They include providing basic and comprehensive emergency obstetric care, the use of antenatal steroids for women in preterm labour, training in the immediate care of the newborn and neonatal resuscitation and post-resuscitation management and ongoing neonatal care (Velaphi & Rhouda 2012).

Postnatal women are escorted by primary caregivers at the health care facility during delivery to assist the postnatal women and the neonates while discharged for home. The intervention was to continue with the postnatal care to the postnatal women and the neonates immediately after the discharge period. Consequently, primary caregivers are not involved by midwives in the postnatal care activities. Midwives only cater to the postnatal women and neonates. Despite the interventions that are in place, the researcher observed that neonates continue to die in South Africa during the early postnatal period. The South African Nursing Council (r. 2488) has regulations that guide midwives on the activities to carry on immediately after the delivery of the baby (*Nursing Act, 33 of 2005*). Based on the stipulated regulations, primary caregivers were not included in those activities; hence, they were neglected by postnatal midwives during the early discharge period (Buek, Cortez & Mandell 2021). Primary caregivers were also responsible for the postnatal care activities at home for the promotion of the postnatal women and neonates’ health as they were the only people responsible for the postnatal care activities at home until the period of 6 weeks. Midwives’ relationship with primary caregivers was not evident during the early discharge period; hence, they were seldom included in the health promotion and counselling opportunities. This was supported by Pace, Crowther and Lau (2022), displaying the lack of supportive relationships between midwives and primary caregivers. Primary caregivers also felt neglected during the early discharge period as the attending midwives did not talk to them nor listen to their complaints at the healthcare facilities (Hanrahan 2021).

According to Gogia and Sachdev (2016), home-based neonatal care was conducted in low- and middle-income countries in South Asia settings by community healthcare workers for a reduction in neonatal and perinatal mortality. The countries were faced with high neonatal mortality rates and poor access to health facility-based care. In the rural areas of Limpopo province, there was no community-based postnatal care model developed for primary caregivers (Shirindza, Malwela & Maputle 2021). Therefore, the article calls for the adoption of a community-based postnatal care model for the management of neonates within the rural areas of Limpopo province.

## Research methods and design

A community-based postnatal care model for the management of neonates in the rural areas of Limpopo province was developed in four phases following the objectives of the study, namely a description of primary caregiver’s experiences of continuity of care and that of midwives in managing postnatal women and neonates during the early discharge period, concept analysis of community-based care, model development and an evaluation phase. The identified concepts and sub-concepts were classified and developed into a conceptual model within the six elements of the practice theory (Dickoff, James & Wiedenbach 1968). These elements are recipients, agents, context, procedure, purpose and dynamics. The relational statements derived after the conceptualisation of each of the six concepts were inferred through the process of deductive analysis and synthesis. The development of a community-based postnatal model was inclusive of the consumer’s suggestions and contained six components, namely, goals, concepts, definitions, relationships, structures and assumptions, as outlined in Chinn and Kramer (eds. 2018). The model was evaluated following Chinn and Kramer’s method and refined by experts in midwifery practice. Limitations were recognised and recommendations were made.

### Study population and sampling strategy

The population in this study consisted of all primary caregivers who attended to the postnatal women and neonates at the health care facilities during the discharge period and the midwives who were providing postnatal care in the health care facilities within the health centers of the three selected districts of Mopani, Sekhukhune and Vhembe district, Limpopo province. Non-probability, convenience, and purposive sampling of primary caregivers and midwives were used in the study. Both primary caregivers and midwives were sampled based on their availability and having consented to participate in the study. In this study, 20 primary caregivers and 100 midwives were sampled when data saturation was achieved.

The non-probability sampling of facilities was based on the high number of maternal and neonatal deaths in the areas identified. The healthcare facilities that were selected were the 10 health centers from the three selected districts in the Limpopo province. The researcher selected available primary caregivers and midwives at the identified health centers who met the inclusion criteria. The primary caregivers were interviewed in the cubicles that were not used by midwives within 6 h after delivery, to enable them to describe their experiences of postnatal care during the early discharge period at home. The midwives were given questionnaires to complete during the early discharge period. All the midwives who worked with the postnatal women and agreed to participate in the study were sampled. Midwives were given a checklist that was completed in the postnatal cubicles, for not more than 45 min and within 6 h after the discharge of a postnatal woman and her neonate. In the qualitative research design, the adequacy of the sample was attained when sufficient data had been collected until data saturation and variation were accounted for and understood.

### Data collection

The approval for the conduct of the study was obtained from the relevant authorities as well as the participants who agreed to participate. The study was conducted in the postnatal units of the health centers within the three selected healthcare districts of Limpopo province. A pilot study was conducted in the postnatal unit within the health care facility where the researcher had been working and was conducted on a limited number of participants from the same population to detect the possible flaws in the data-collection instruments and to investigate the feasibility of the proposed study.

The researcher used different data-collection methods to maximise the quality of data as well as the reduction of the chances of bias. The researcher used in-depth one-to-one interviews to collect data from primary caregivers to gather detailed information about the management of neonates during the early discharge period and distributed questionnaires to midwives regarding the facilitators and barriers encountered during the early discharge period. Interviews with primary caregivers were conducted in the cubicles or duty rooms as arranged with midwives. The interviews were conducted using Xitsonga, Tshivenda or Sepedi based on the language used by primary caregivers. The researcher employed experts to interview primary caregivers based on language barriers for correct data. The interviews were tape-recorded and the confidentiality of the data obtained, and the anonymity of the participants were adhered to. The tapes were numbered, and the participants’ names were not mentioned. During the interview process, communication techniques such as paraphrasing, summarising, probing and listening were used to obtain necessary information from the participants (Brink, Van der Walt & Van Rensburg 2018). The researcher used a Likert scale to collect data from midwives which took only 30 min with the help of an assistant researcher who assisted the researcher with the collection of questionnaires to the researcher.

### Data analysis

The informative data from the one-to-one individual interviews were analysed qualitatively using Tesch’s open coding system (Creswell & Creswell 2018). The independent coder was also requested to analyse data based on the experiences in qualitative data analysis. The two analysis methods ensured the trustworthiness of the study. Quantitative data were analysed through descriptive statistics utilising the computerised Statistical Package for the Social Science (SPSS) program version 25. Data collected from midwives were analysed using descriptive statistics that assisted the researcher in describing and explaining the data.

## Trustworthiness

Trustworthiness as a method of establishing rigour was used to ascertain that the outcomes of the study be trusted and that the study be reproducible (Burns & Grove 2011). The researcher employed four criteria of trustworthiness, namely credibility, transferability, dependability and applicability to establish the trustworthiness of the study as cited in Lincoln and Guba (1985). The following are the measures described to establish trustworthiness that are discussed further in the text.

### Credibility

Credibility in this study was ensured to ascertain confidence in the truth of the findings from the participants and the context in which the study was undertaken. The activities that increased credible findings were prolonged engagement, peer debriefing, triangulation, member checking and negative case analysis.

### Dependability

In this study, the stability or reliability of the study was achieved through an audit trial. The reviewer examined the detailed records of all the documentation of critical incidents and the products such as findings, interpretations and recommendations and attested that the data were supported by the findings.

### Transferability

The researcher attained transferability through the thick description of the methodology selected that provided thick descriptive data to assist the readers in evaluating the applicability of the information to other contexts. Purposive sampling was also used to maximise the range of specific information that could be obtained from the contexts, by purposely selecting locations as well as participants with different characteristics within the study population.

### Confirmability

The confirmability of the study was tested through the involvement of the independent coder to analyse the transcripts, reviewing the raw data and tape recorder, the written field notes, results, and the documents that were analysed independently. The independent coder verified the representativeness of the collected data to check whether the researcher had interviewed the relevant categories of participants that gave a clear picture of the study project.

### Ethical considerations

The quality of the study was ensured by adhering to the highest possible standards of research through accountability as well as the ability to execute the research process. Ethical standards were ensured by obtaining ethical clearance (Ref: SHS/19/PDC/07/2904), from the University of Venda Ethics Committee, permission to conduct the study from the Limpopo Provincial Department of Health, the District Managers of the three selected districts and the participants from the three ethnic groups. Each participant was provided with relevant and correct information regarding the indications for participation in the study. The participants’ confidentiality and anonymity were ensured by protecting their privacy, identity and dignity. The right to self-determination was protected by obtaining informed written consent from participants for participation in the study and also by informing participants of their rights to withdraw from the study without any penalty.

## Discussion

The results from the experiences of primary caregivers and the facilitators and barriers for midwives during the early discharge period revealed three themes, seven sub-themes and categories. The themes were classified according to their numbers and the following are the categories of the themes:

- Traditional knowledge of primary caregivers about postnatal care
- Performance of cultural practices during the continuity of postnatal care
- Skills and competencies of primary caregivers in providing continuity of postnatal care
- Non-involvement of primary caregivers
- Good midwives and postnatal women’s relationship
- Perception of information dissemination

### Description of community-based postnatal care model

Following the study ‘Primary caregivers’ experiences of management of neonates in the rural area of Limpopo province’ (Shirindza et al. 2021) and the study ‘Facilitators and barriers for midwives for managing postnatal women and neonates during the early discharge postnatal period’ (not discussed here), the community-based postnatal care model was developed. The development of the community-based postnatal care model contained the following six components: (1) goals, (2) concepts, (3) definitions, (4) relationships, (5) structures and (6) assumptions, as outlined in Chinn and Jacobs (1987).

### Theory components

Based on the study of ‘primary caregivers’ experiences during the early discharge postnatal period’ and the ‘midwives’ facilitators and barriers during the early discharge postnatal period’ (Shirindza et al. 2021), which was not discussed in the article here, the community-based postnatal care model was developed. The model developed contained six elements, namely, goals, concepts, definitions, relationships, structures and assumptions (Chinn & Jacobs 1987).

### Concepts

Concept analysis was done from the analysis of data and ‘community-based postnatal care’ emerged as a central concept (Shirindza et al. 2021). Concept analysis was done following Walker and Avant (2005). The results of the concept analysis gave a clear direction on the classification of concepts and sub-concepts for the development of the model (Shirindza et al. 2021). The identified concepts and subconcepts were classified and developed into a model as outlined by Dickoff et al.’s survey list (1968). The elements are: (1) recipient, (2) agent, (3) context, (4) procedure, (5) purpose and (6) dynamic. After conceptualisation of the identified concepts of the practice elements of the theory as outlined by Dickoff et al. (1968), the relational statements were inferred through deductive analysis and synthesis. The relational statements formed the basis for the development of the model, containing six components as outlined by Chinn and Jacobs (1987), namely goals, concepts, definitions, relationships, structure and assumptions. The model was later evaluated following Chinn and Kramer (1995) and refined by experts in midwifery and model creation. The definitions of ‘community-based’ postnatal care were examined during concept analysis but were not discussed within this article.

### Relationships

The definition of the relationship was adopted through the designing of the relational statements according to the synthesis of the existing definition of ‘community-based’ postnatal care. Synthesis is the process that strategises the creation of something new from the already available data. ‘Community-based’ postnatal care was synthesised by the researcher within the context of the study and also adapted for this study.

### Structure

**FIGURE 1:**
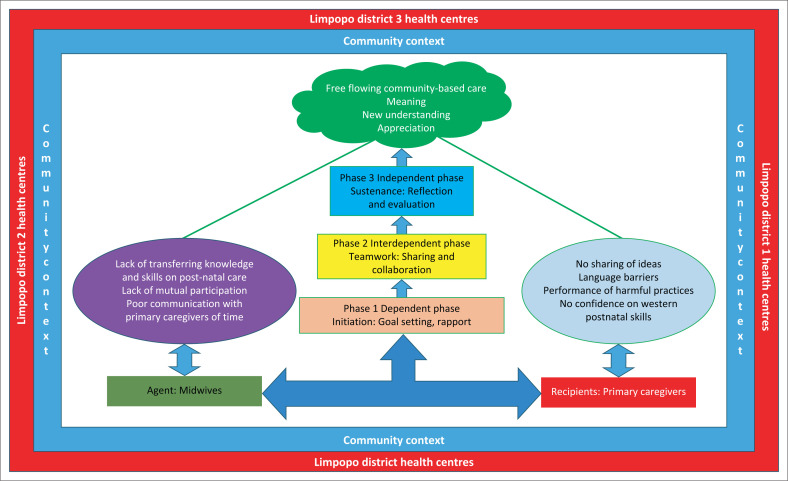
A community-based postnatal care model.

Conceptual model: After the synthesis process, the conceptual model was developed using Dickoff et al.’s (1968) survey list that encompasses the agent, recipient, procedure, context, dynamics and terminus (Table 1 and Figure 1).

**TABLE 1:** Conceptual model.

Context	The context was the health care context and the community context within the selected districts of the Mopani, Sekhukhune and Vhembe in Limpopo province where the interaction took place between primary caregivers and the midwives during the early discharge period.
Recipient: Primary caregivers	She is a person identified under her caring for the postnatal woman and her neonate at home. The primary caregivers were found to be independent of the midwives. This was evidenced by the dependency on traditional and cultural postnatal skills as well as their non-involvement during the early discharge period by midwives.
Agent: Midwives	The midwives are professionals with specific skills who interact with primary caregivers with understanding, knowledge, skills and abilities to add value, quality and improvement to the rendering of postnatal care activities.
Dynamics	The dynamics are the power sources that would facilitate the process of mutual participation between primary caregivers and midwives. Lack of power, language barriers, no sharing of ideas or openness and no honesty as they were practicing traditional postnatal care skills.
Procedure: Mutual participation	Mutual participation during postnatal care is the cornerstone of community-based care. It is a reciprocal process that facilitates negotiating goals between midwives and primary caregivers and can be achieved through appropriate health education programs. Open communication with goal setting at the onset, establishing a rapport with primary caregivers and accepting other parties’ opinions without advocating for themselves.
Terminus	The outcome of the activity would be the achievement of meaningful participation where primary caregivers and midwives in rural areas engage with each other in community-based postnatal care and have primary caregivers that are well informed about the postnatal care activities that are not harmful that may enable them to make responsible decisions.

*Source:* Maputle, M.S., 2010, ‘A woman-centred childbirth model’, *Health Gesondheid* 15(1), 450. https://doi.org/10.4102/hsag.v15i1.450

### Process of the model

The model depicts that through the procedure of providing community-based postnatal care practice, three phases are involved.

#### Phase 1: Dependent phase

Initiation, goal setting and establishing rapport with the engaged parties.

This is the initial stage that involves the agent and the recipient. During this phase, the agents and the recipients re-examine the values, meanings and assumptions they already have about community-based postnatal care skills. The researcher assumes that the rural cultural context in which community-based postnatal care is supposed to take place influences how primary caregivers can initiate community-based postnatal skills. This means that the agent and the recipient should re-examine their postnatal caring skills through clarification of values to create awareness to bracket them and be open to new information regarding traditional postnatal skills.

Both primary caregivers and midwives are given time to re-examine themselves as individuals and in a group as a group of midwives and primary caregivers. The groups are given time to present the outcomes of the examinations and all participants are allowed free discussions about the issues presented and assisted to overcome obstacles to community-based postnatal care that arose from the presentation. Individuals with information that needs confidentiality should be given time to be handled as such and consulted confidently.

The initial phase begins with the initiation and goal setting for the establishment of rapport for primary caregivers to accept meaningful community-based postnatal care. The facilitator engages all participants in the identification of dynamics that could be involved during goal setting regarding acceptable community-based postnatal care skills. A decision about the dynamics is therefore encouraged. The dynamics will also emerge from both primary caregivers and the midwives during the clarification of values. The participants also come up with strategies to deal with such challenges or dynamics to engage in community-based postnatal care.

#### Phase 2: Interdependent phase

This is the second phase that involves mutual participation. This is the stage in which the primary caregivers and midwives interact for the exchange of information. During this phase, both the midwives and primary caregivers engage in participatory equal discussions regarding postnatal skills at home. Facilitation of mutual participation (interdependent phase) entailed primary caregivers and midwives who agreed on the plan and documented all aspects related to community-based postnatal care.

As a point of departure, the researcher gives the participants the definition of community-based postnatal care as ‘the early discharge postnatal care activities that are performed to postnatal women and neonates in the community context’. This means providing postnatal care activities free from infections, postnatal complications and good quality health status. The questions regarding the quality of postnatal care skills are addressed. Participants are given time to reflect on their outputs and to discuss measures to correct the differences related to the postnatal caring activities and also involve them in what they think might improve. Teamwork, mutual trust, power differences, honesty and power indifferences should be taken into account for interdependency to be well established.

#### Phase 3: Outcomes following the implementation of the plan (independent phase)

During this phase, the participants examine their unexamined assumptions for self-destruction. During this phase the participants are not supposed to advocate for their perspectives, rather they have to accept scrutiny by others and allow them to be evaluated. This is the stage where the participants are to accept new things and discard the old way of doing things. It is during this phase where the recipients and the agents have to engage with each other, thus taking the correct skills of postnatal care activities and discarding the traditional postnatal care skills.

The discussions of the empirical findings of the primary caregivers’ experiences of early discharge postnatal care and the facilitators and barriers of midwives revealed that there were similarities and differences regarding the postnatal caring activities including cord care, breastfeeding and breast care, eye care, postpartum haemorrhage management and prevention of maternal and neonatal sepsis.

The results indicated that mutual participation was not visible between midwives and primary caregivers during the early discharge period.

Primary caregivers relied on traditional knowledge and the performance of harmful practices during the early discharge period. They had no confidence in the use of Western postnatal caring skills because of a lack of support from midwives. Midwives did not even display an agreement with the traditional knowledge and cultural practices displayed by primary caregivers.

*Facilitation of mutual participation:* In mutual participation, midwives displayed limited feelings of deep admiration for the cultural practices performed by primary caregivers during the early discharge period. Primary caregivers were not even involved during the early discharge period; hence the midwives were concentrating on the postnatal women and the neonates without involving primary caregivers. There was a poor relationship between midwives and primary caregivers as evidenced by poor communication related to the language barrier. This contributed to the primary caregivers continuing with the performance of traditional practices as they were not even guided by midwives. Midwives also had no compassion for the sufferings of primary caregivers as they were not even admired by midwives during the visitation at the health care facilities. Limited opportunities created by midwives created powerlessness for primary caregivers as evidenced by a lack of agreement regarding their traditional activities and general disagreement on cultural practices between midwives and primary caregivers when assisting the postnatal woman and neonates. Primary caregivers were also faced with language barriers during the early discharge period. Midwives did not even care about the primary caregiver’s language problems; instead, they continued with postnatal discharge activities without involving them hence midwives displayed negative attitudes towards primary caregivers.

*Information dissemination:* The actions taken by midwives were to promote, support and respect the values and preferences of primary caregivers regarding postnatal caring skills as long as they were not harmful to the mother and baby. However, midwives did not even disseminate good information to primary caregivers during the early discharge period for continuity of care at home. This shows that midwives had limited communication with primary caregivers regarding the postnatal skills to be considered as the members of the family for the postnatal women and the neonates.

#### Interventions to enhance mutual participation during the community-based postnatal care (interdependent phase)

Teamwork is the strategy that would be employed by midwives to involve primary caregivers in the management of neonates at the community level after discharge. Through teamwork and collaboration on the agreed postnatal care activities, both two parties would be able to act positively for the enhancement of mutual participation.

According to the Bill of Rights, as stipulated within Chapter 2 of the Constitution of the Republic of South Africa (1996), Chapter 2 of the *Constitution of the Republic South Africa* (1996), every individual member as a citizen has a right to access information that will benefit them as citizens of South African (Hames 2013). This means that primary caregivers have the right to access information from midwives, which they require for community-based postnatal care at home or within the community context. The information that they will receive from midwives will assist them in bringing about positive changes to the harmful traditional practices if the midwives responsible could give them effective and sufficient data regarding the management of neonates during the early postnatal period.

Both midwives and primary caregivers should begin with communication from the onset for initiation and goal setting to achieve the desired and best outcomes. To achieve the goals, primary caregivers should be considered by midwives in decision-making together so that they can bring out positive changes in the outcomes of the postnatal women and neonates without some discomforts and risks. Primary caregivers should be able to participate mutually with midwives during the early discharge period by forming teams, collaborating effectively and giving each other health information and the risks associated with traditional and cultural practices. Primary caregivers should be given more power to verbalise their fears and concerns regarding the cultural practices, provided with the state of independence so that they can understand that they have the power to communicate with midwives for the correct action on postnatal activities while at home. This will also demonstrate to the primary caregivers that they also have an enormous contribution to the caring practices that are acceptable to both the midwives and the postnatal women and the neonates.

During this phase, midwives should continue with the establishment of training programs related to the care of neonates during the community meetings to create a positive relationship with primary caregivers. The established training programs within the community will provide primary caregivers with the necessary information that will also assist them in challenging postnatal complications in the absence of skilled midwives. This strategy will assist primary caregivers in developing confidence and self-esteem in the postnatal skills rendered to the postnatal women and neonates.

Midwives are important members in introducing team integration with stakeholders within the community to maintain connection and support from them. Because community stakeholders are social, creative and well-connected individuals within social networks, it would be very effective to bring the type of facilitators that will add value to the teamwork and collaborations with midwives and primary caregivers for the management of neonates in the community context (Lebese 2009). It will be of great value to invite the important stakeholders in the community who have a close understanding of the needs of postnatal women during the early discharge period. The teamwork between the stakeholders and the midwives will strengthen the support and the ongoing support between midwives and primary caregivers regarding the management of neonates in the rural community.

Primary caregivers and community stakeholders should also be involved or included in consistently organised case management meetings with midwives to understand all the critical issues that postnatal women and neonates are faced with. This will enable the team of midwives and primary caregivers to act jointly on the steps to be taken to correct or help build up cases of traditional postnatal care practices performed by primary caregivers during the postnatal period.

Setting expectations after the development of the model plays an important role in the research as it allows the team of model users what to expect from the model designer. The outcomes of the community-based postnatal model demonstrate the specific behaviours or results that are expected between the primary caregivers and midwives after the implementation of the model. The primary caregivers are expected to provide the postnatal women and neonates with a nutritious diet and healthier foods during the postnatal period. The woman is no longer expected to eat a diet that is locally available and not suitable for her and the neonates. All the local practices and taboos related to postnatal women and neonates should be abolished and postnatal women and neonates should have access to good nutrition, good perineal hygiene, proper umbilical cord care, promotion of breastfeeding, delay in the establishment of solid foods to the neonate, the avoidance of all the harmful practices that are performed traditionally such as the application of cow dung to the umbilical cord of a neonate for healing process, all unhygienic practices should be abolished.

There is enough information revealing that midwives wish to involve primary caregivers in mutual participation. Community-based postnatal care model will take time to develop, therefore, primary caregivers need to be accommodated and accepted as human beings until they are perfect in the community-based postnatal skills.

### Evaluation of the model

According to Chinn and Kramer (2008), empirical knowledge can be authenticated to ensure that the researcher is honest with himself and others. The authenticity of the model was performed through confirmation or validation. The theory validating research has a specific meaning for validation and would imply specific methods to be used. Chinn and Kramer (2008) indicated that special methods are designed to determine the accuracy of the theory in depicting the empirical phenomena and their relationships. The theoretic statements can be translated into questions or hypotheses. The theory-validating research further reflected that no one study can test the entity of the theory. Therefore, it becomes evident that the theory validating research usually considers a deductive approach rather than an inductive approach.

In this study, the guidelines for the critical reflection of the theory were used to test the model (Chinn & Kramer 1995). The model was further tested by experts in midwifery such as registered midwives, operational managers and district maternal and child health care managers in the three selected districts of Limpopo province. A survey was conducted to check whether the identified challenges in community-based care could be addressed by the developed model thus bringing a reduction in maternal and neonatal mortality rate.

According to Chinn and Jacobs (1987), there are factors to take into account before application of the model:

- Theory–goal and practice–goal relationship, meaning that the goals should be clear to both groups to achieve a higher level of performance in community-based postnatal care, thus reducing neonatal deaths
- Situational factors, such as lack of education by primary caregivers, and observing the external influence can impact the community-based postnatal care practices by primary caregivers
- Theory variables and practice variables as there are variables that can hamper the application of the model such as language barriers and age of the primary caregivers in this case
- Nursing actions and research evidence, identifying if the model could provide holistic, quality community-based postnatal care that is up-to-date rather than the traditional methods or personal beliefs from primary caregivers.

These prerequisites were considered in the study to make a sound judgment on the application of the model. The group of experts in midwifery reached an agreement that the model would be of importance in community-based postnatal care practice. The model was presented locally with research experts who evaluated and made valuable contributions to the model.

### Limitations of the study

The model was not piloted as this was out of the scope of the study. The empirical data from the participants were restricted to only three selected districts of the province; hence, the findings reported only the experiences, barriers and facilitators within the three selected districts in the province. The model would therefore be useful in different community settings in African countries. Therefore, the community-based postnatal care model still needs to be implemented practically.

### Recommendations

The recommendations are made for all maternity facilities and communities where there is a lack of interaction between the primary caregivers and the midwives regarding the management of neonates. Limited participation and dependency may be experienced by primary caregivers in various healthcare facilities and communities wherever the family member is involved in interaction with midwives. The model is aimed at enhancing the mutual participation between primary caregivers and midwives in the provisioning of community-based postnatal care to manage neonates in rural areas.

## Conclusion

The study was undertaken to construct a community-based postnatal care model. The aim was to share the community-based postnatal care model that could be used to assist primary caregivers and midwives in the management of neonates during the early postnatal care period. The constructed model would be used to enhance its implementation in the health care facilities during early discharge postnatal care.
